# Sequence-Based Predictions of Lipooligosaccharide Diversity in the *Neisseriaceae* and Their Implication in Pathogenicity

**DOI:** 10.1371/journal.pone.0018923

**Published:** 2011-04-18

**Authors:** Daniel C. Stein, Clinton J. Miller, Senthil V. Bhoopalan, Daniel D. Sommer

**Affiliations:** 1 Department of Cell Biology and Molecular Genetics, University of Maryland, College Park, Maryland, United States of America; 2 Institute for Advanced Computer Studies, University of Maryland, College Park, Maryland, United States of America; Indian Institute of Science, India

## Abstract

Endotoxin [Lipopolysaccharide (LPS)/Lipooligosaccharide (LOS)] is an important virulence determinant in gram negative bacteria. While the genetic basis of endotoxin production and its role in disease in the pathogenic *Neisseria* has been extensively studied, little research has focused on the genetic basis of LOS biosynthesis in commensal *Neisseria*. We determined the genomic sequences of a variety of commensal *Neisseria* strains, and compared these sequences, along with other genomic sequences available from various sequencing centers from commensal and pathogenic strains, to identify genes involved in LOS biosynthesis. This allowed us to make structural predictions as to differences in LOS seen between commensal and pathogenic strains. We determined that all neisserial strains possess a conserved set of genes needed to make a common 3-Deoxy-D-manno-octulosonic acid -heptose core structure. However, significant genomic differences in glycosyl transferase genes support the published literature indicating compositional differences in the terminal oligosaccharides. This was most pronounced in commensal strains that were distally related to the gonococcus and meningococcus. These strains possessed a homolog of heptosyltransferase III, suggesting that they differ from the pathogenic strains by the presence a third heptose. Furthermore, most commensal strains possess homologs of genes needed to synthesize lipopolysaccharide (LPS). *N. cinerea*, a commensal species that is highly related to the gonococcus has lost the ability to make sialyltransferase. Overall genomic comparisons of various neisserial strains indicate that significant recombination/genetic acquisition/loss has occurred within the genus, and this muddles proper speciation.

## Introduction

Lipopolysaccharide (LPS) or its naturally occurring variant lipooligosaccharide (LOS) is an essential outer membrane component of gram negative bacteria (see [1] for a recent review). LOS differs from LPS in that LOS naturally lacks the O-repeating carbohydrate polymer. Both LOS and LPS contain a core polysaccharide that is covalently linked to lipid A. Most enzymes responsible for the biosynthesis of these molecules have been identified, and the genes can be identified in most gram-negative bacteria based on genetic homology to biochemically characterized proteins. The structure of LPS differs from one bacterium to another, with its composition defined by the biochemistry of the various proteins.

**Figure 1:**
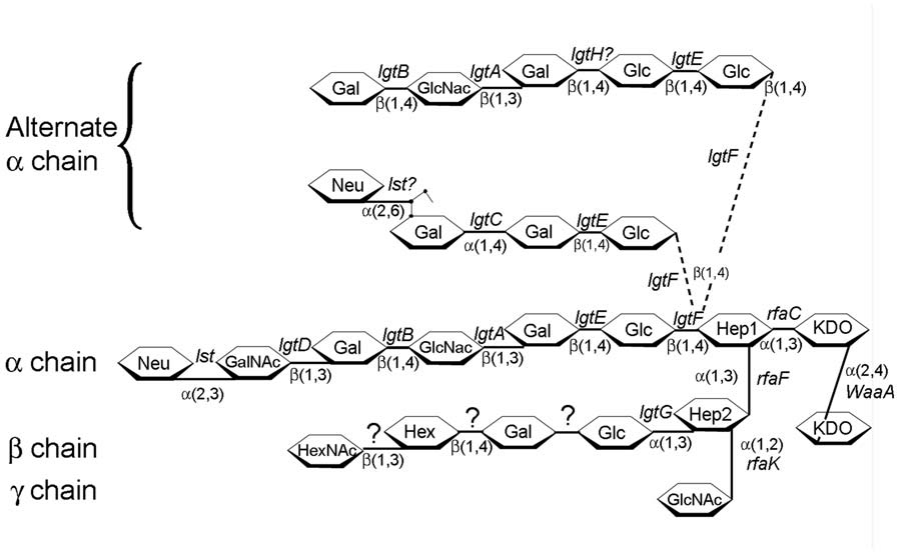
LOS structures found in pathogenic *Neisseria* strains. The structure of LOS presented in this figure is a composite derived from several published structures of neisserial LOS [5], [6], [8], [15], [28], [30], [44], [49], [59], [60], [61].

In the *Neisseriaceae*, LOS expression undergoes high frequency structural variation [2], with the variation in the polysaccharide being implicated in pathogenesis [3]. It has been extensively characterized through a variety of structural [4], [5], [6], [7], [8], biochemical [9], [10], [11], [12], [13], [14] and genetic methods [15], [16], [17], [18], [19]. Its impact on neisserial biology is broad, affecting both the organisms basic biological properties [20] and the influence the organism has on the host [21], [22], [23], [24], [25]. Neisserial LOS is composed of lipid A, which anchors the oligosaccharide chains to the membrane, an inner core composed of two 3-Deoxy-D-manno-octulosonic acid (KDO) residues and two heptose residues, and an oligosaccharide extension from the inner core. Inner core residues connect the outer core to the lipid A anchor. The data in Figure 1 represent a structural schematic and genetic summary of variation in carbohydrate structures that have been seen in LOS isolated from pathogenic strains. In *N. gonorrhoeae* and *N. meningitidis*, the genes responsible for the production of LOS are found at seven genomic locations: *kdtA*, containing the gene needed to add the two KDOs onto the lipid A core [26]; *rfaC* whose product adds a heptose residue to KDO [27]; *lgtF* and *rfaK*, which encode for genes responsible for the initiation of the α chain and synthesis of the γ chain [28]; *rfaF*, adds a second heptose residue to the first heptose [29]; *lgtABCDE*, which encodes for the bulk of the genes responsible for the extension of the α chain [30]; *lgtG*, which encodes the gene responsible for the synthesis of the β chain [16]; and *lst*, which can add a sialic acid cap to the oligosaccharide chain [31]. To date, no genes involved in LOS biosynthesis in commensal organisms have been described.

Commensal *Neisseria* have been shown to serve as a source of genetic diversity in the genus [32], [33], [34]. Genomic studies on *N. lactamica* have found regions of horizontally acquired DNA and the presence of many *N. meningitidis* virulence-associated genes [35], [36]. A few studies have analyzed the LOS produced by the commensal *Neisseria* and the data indicate that LOS heterogeneity extends beyond what is seen in the gonococcus and meningococcus [37], [38]. These findings suggest that alternative LOS structures are present in commensal *Neisseria*. In this study, we compared the genomic content of various commensal and pathogenic *Neisseria* strains, with respect to LOS biosynthesis in order to assess the degree of genomic variability in LOS biosynthetic genes. Given the role of transformation in driving genomic variability and the nature of the flanking genes in most LOS biosynthetic loci in the pathogens, we hypothesized that we could use DNA sequence conservation in genes flanking LOS biosynthesis genes to identify regions important in LOS biosynthesis in commensal organisms. This paper represents a genomic analysis of commensal neisserial DNA sequences derived in our laboratory and in published databases.

## Results

### Presence of KDO transferase (WaaA)

**Figure 2:**
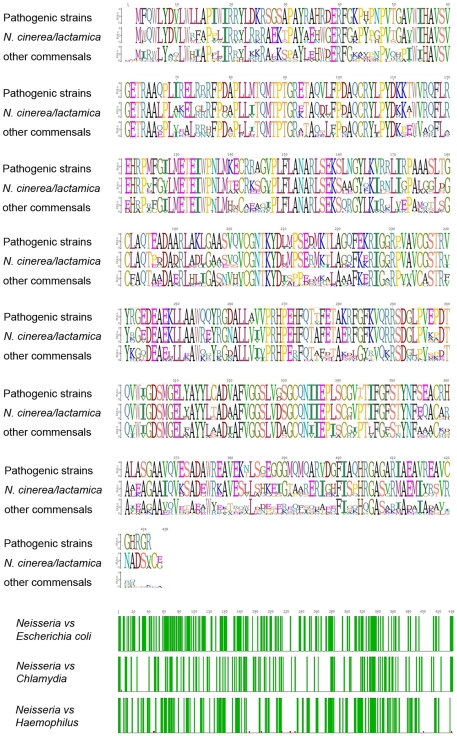
Analysis of KDO transferases from various *Neisseria* strains. Panel A is a Sequence Logo created using the predicted amino acid sequences of *rfaC* from various neisserial strains. The DNA sequences used for this analysis were *N. gonorrhoeae* strains; 1291, 35/02, DGI18, DGI2, F62, FA19, FA6140, MS11, PID1, PID18, PID24-1, PID332, SK-92-679 and SK-93-1035 [sequences from the Broad Institute], FA1090 (accession number AE004969); and NCCP11945 (Accession number CP001050). The DNA sequences for *N. meningitidis* were NS44 (DCS, unpublished sequence), 053442 (accession number CP000381), FAM18 (accession number AM421808), 22491 (accession number AL157959-1), MC58 (accession number AE002098.2), alpha 14 (accession number AM889136.1). Commensal Neisserial strain DNA sequences were: *Neisseria cinerea* ATCC 14685 (accession number ACDY00000000); *N. elongata* subsp. glycolytica ATCC 29315 (accession number ADBF00000000); *N. flavescens* strain SK114 (accession number ACQV00000000) and strain NRL30031/H210 (accession number ACEN00000000); *N. lactamica* ATCC 23970 (accession number ACEQ00000000), Y92-1009 (Project ID: 50739), ST-640 (Project ID: 13472), and strain NS19 (DCS, unpublished sequence); *N. mucosa* ATCC 25996 (accession number ACDX00000000) and C102 (Project ID: 38747); *N. polysaccharea* ATCC 43768 (accession number ADBE00000000) and NS342 (DCS, unpublished sequence); *N. sicca* ATCC 29256 (accession number ACKO00000000), 4320 (DCS, unpublished sequence) and DS1 (DCS, unpublished sequence); *Neisseria sp.* oral taxon 014 str. F0314 (Project ID: 49701); and *N. subflava* NJ9703 (accession number ACEO00000000). The DNA sequences for *Haemophilus influenzae* were: Rd KW20 (accession number L42023), 86-028NP (accession number CP000057), PittEE (accession number CP000671) and PittGG (accession number CP000672. The DNA sequences for *Chlamydia trachomatis* were: strain 434/Bu (accession number B0B9V8) and strain A/HAR-13 (accession number Q3KMF4).

The key sugar that connects core sugars to lipid A is KDO. KDO transferases are multifunctional enzymes that are able to transfer several KDO residues from CMP-KDO to different precursor molecules forming different linkages. The DNA sequence of *waaA* from *N. meningitidis* [39] was used to search the various databases containing both pathogenic and commensal genomic DNA sequences. The data in Figure 2, panel A indicate that all strains examined contained a gene that would likely encode this KDO transferase. The degree of amino acid sequence identity in the pathogenic strains was quite high, exceeding 98% identity in all strains. The two organisms that are closely related to these pathogenic strains, *N. cinerea* and *N. lactamica* showed an intermediate level of homology. However, the homology in all of the commensal strains exceeded 75% identity.

The biochemical specificity of WaaA is quite diverse: in *Haemophilus influenzae*, it is monofunctional [40]; in *Escherichia coli* it is bifunctional [41]; while in *Chlamydiaceae* it is at least trifunctional [42]. We used the WaaA sequence of *E. coli* or *H. influenzae* to search the available translated neisserial DNA sequences and found that the these proteins were very similar, with homology between the proteins seen across the entire protein sequence. Searches using the *Chlamydia trachomatis* WaaA revealed that this protein was much less similar to the neisserial WaaA, with homologies strongest in the middle of the protein.

In *Haemophilus spp.*, the second KDO is functionally replaced by a phosphate through the action of a kinase, KdkA [43]. We searched the neisserial genomes for the presence of *kdkA*. Since we were unable to identify any ORFs with significant homology to KdkA, we concluded that the neisserial WaaA is a bifunctional enzyme. This would indicate that LOS isolated from commensal and pathogenic *Neisseria* strains should all possess two KDO residues. This is supported by all structural data on neisserial LOSs, which indicate that they all possess two KDO residues [7], [44], [45], [46], [47], [48], [49].

### Presence of heptosyl transferases

**Figure 3:**
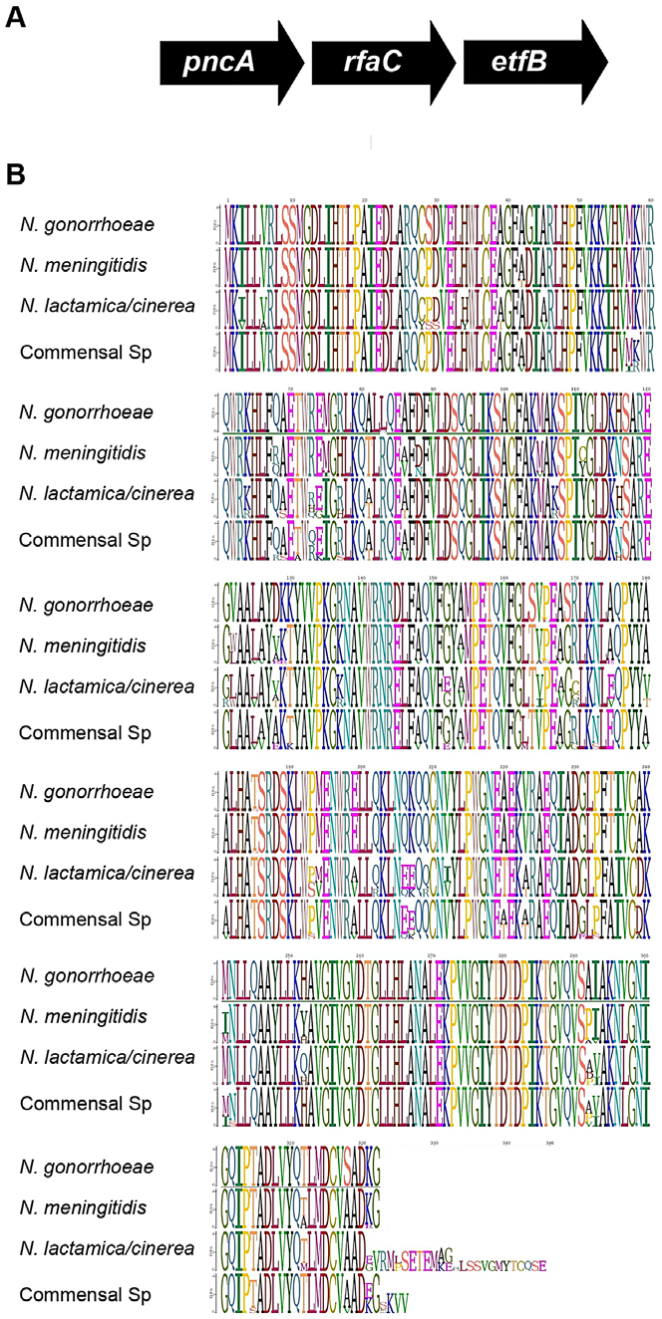
Analysis of the *rfaC* region in the *Neisseriaceae*. Panel A. Genomic organization of the *rfaC* region. The gene *pncA* encodes pyrazinamidase/nicotinamidase, and the gene etfB encodes an electron transfer protein. Panel B is a Sequence Logo alignment of the *rfaC* gene from the various sequenced neisserial strains.

RfaC is required to add heptose I onto KDO in most microorganisms. We used the DNA sequence of *rfaC* [27] to search the various neisserial genomes for the presence of *rfaC*. The data in Figure 3 indicate that all neisserial strains possess an *rfaC* homolog. The genomic organization of *rfaC* was conserved in all neisserial species with the flanking genes sharing significant nucleotide similarities (Figure 3, panel A), even though the ascribed functions are unrelated to LOS biosynthesis.

In the gonococcus, the predicted amino acid sequence of *rfaC* was virtually identical in all 16 strains examined, with a total of three differences observed, one each in three strains (Figure 3, panel B). The amino acid sequences of the meningococcus differed significantly more, with 27 variable residues found in only eight strains. Variation in protein sequence was even more diverse when we analyzed the *rfaC* sequence of *N. lactamica* or other commensal organisms. While we observed significant amino acid sequence variability, we also noted that the terminal sequence of the protein varied significantly, due to mutations in the stop codon found in the pathogens, with readthrough adding between 4 and 16 amino acids to the protein. It is unclear why the sequence is so conserved in the gonococcus, while the variation in other strains is more consistent with what is seen with most LOS biosynthetic genes.

**Figure 4:**
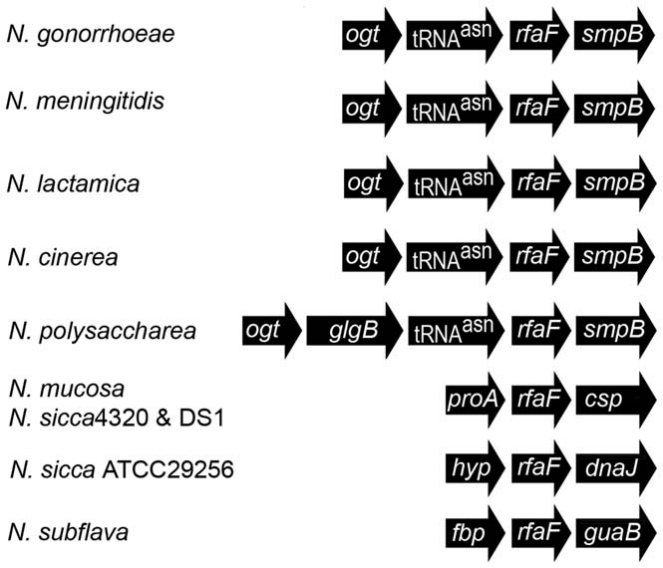
Analysis of the *rfaF* region in the *Neisseriaceae*. The flanking genes encode: *ogt*, O^6^-methylguanine-DNA methyltransferase; *smpB*, *RNA-binding protein*, *proA*, gamma-glutamylphosphate reductase, *csp*, cold shock protein; *hyp*, hypothetical protein; *dnaJ*, Heat shock protein; *flp*, recombinase; and *guaB*; inosine 5′-monophosphate dehydrogenase.

In the gonococcus, the gene *rfaF* is responsible for the addition of the second heptose onto heptose 1 [18]. We used the DNA sequence from this gene to search the various nucleotide databases for similar predicted proteins. All strains possessed a predicted protein with greater than 88% similarity across the entire ORF. This indicates that all neisserial LOS should possess at least two heptose residues in their LOS. More interesting was the degree of genomic variability in the regions surrounding *rfaF* (see Figure 4). The genomic organization was identical in all gonococcal and meningococcal strains, as well as in the *N. lactamica* and *N. cinerea*. However, in the commensal strains the organization varied significantly. *N. polysaccharea* possessed an organization similar to that of the pathogens, but had an insertion of a gene with significant homology to *glgB*, an enzyme involved in glucan branching [50]. All of the other commensal strains examined had one of three organizations. Since sequence divergence between the same *Neisseria* spp. is very different at different genetic loci [51], some form of selective pressure has selected for the maintenance of *rfaF*. This demonstrates the importance of the presence of the second heptose in the survival of the organism.

### Elongation of the alpha chain with LgtF

**Figure 5:**
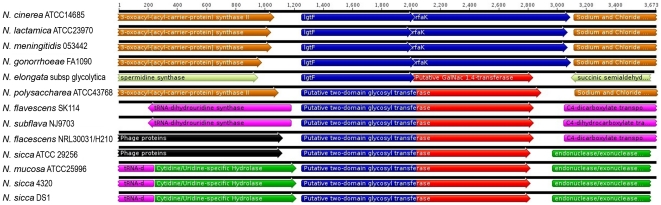
Genomic organization of genes that initiate synthesis of the alpha chain. Accession numbers are the same as in Figure 2.

The pathogenic strains possess the ability to make three alternate alpha chains. The first protein needed to elongate the alpha chain is LgtF. In the pathogens, *lgtF* is part of a two gene cluster that is linked to *rfaK*. We analyzed the genomic organization of this region in the various neisserial species. The data in Figure 5 indicate that the genomic order was conserved in the pathogenic strains, and those closely related to them. In *N. elongata* we were able to identify a clear homolog of *lgtF*. However, the downstream gene, while possessing clear homology to a glycosyl transferase, was different from *rfaK*. While this novel glycosyl transferase was found in all of the other strains examined, it was found as a fusion protein to another glycosyl transferase. In addition, the flanking genomic organization of this novel glycosyl transferase differed significantly in the various strains. These data suggest that the commensal strains elongate their LOS via a sugar other than glucose.

### Presence of gamma chain N-acetylglucosamine transferase

We used the sequence of the FA1090 *rfaK* gene to search the translated genomic sequences of the *Neisseriaceae*. The data in Table 1 demonstrate that all gonococcal and meningococcal strains possessed a protein with 100% homology to the test sequence. In addition, predicted proteins with 95% or greater homology were found in *N. lactamica*, *N. cinerea* and one strain of *N. polysaccharea*. The genomic organization in strains that possessed *lgtF* and *rfaK* were conserved in these strains, containing the same flanking genes. All other commensal strains did not possess the ability to produce proteins with significant homology to RfaK. Database searches of the two flanking genes were identified in all databases, but the two genes did not appear to be linked. This indicates that only the pathogenic neisserial strains, and those closely related possess the ability to synthesize a gamma chain.

**Table 1:** Presence of heptosyl transferase genes.

Strain	*rfaK*	*rfaC*	*waaQ*
Gonococci	+	+	−
Meningococci	+	+	−
*N. cinerea* ATCC 14685	+	+	−
*N. lactamica* ATCC 23970	+	+	−
*N. lactamica* NS19	+	+	−
*N. lactamica* (Sanger)	+	+	−
*N. polysaccharea*	+	+	−
*N. polysaccharea* NS342	−	+	+
*N. mucosa* ATCC 25996	−	+	+
*N. flavescens* NRL30031/H210	−	+	+
*N. sicca* 4320	−	+	+
*N. sicca* DS1	−	+	+
*N. sicca* ATCC 29256	−	+	+
*N. subflava* NJ9703	−	+	+

+ Presence of gene sequence.

− Absence of gene sequence.

The predicted structure of *N. sicca* 4320 core LOS contains three heptose residues [49]. Therefore, we searched the neisserial genomes using the *E. coli* DH1 heptosyl transferase III amino acid sequence. All neisserial strains that lacked RfaK, possessed a gene with a predicted homology of >50% identity with *E. coli* Heptosyl transferase III. When this sequence was used to search the NCBI database using genomic blast, three types of homology with various *Neisseria* strains was observed; High homology with greater than 67% identify and 83% similarity (identifying putative heptosyl transferase III proteins); and two classes of genes with limited homology, which corresponded to heptosyl transferase I and II. From genomic analysis of the inner core region of all neisserial strains, the data indicate that the strains possess two basic core structures; one where they possess a gamma chain N-acetylglucosamine and one that contains a third heptose.

### Modification of neisserial LOS with sialic acid

**Figure 6:**
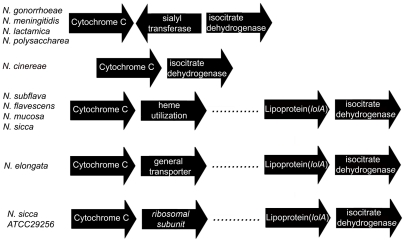
Genomic organization of regions that might contain *lst*. The presence of the *lst* gene in the genomes of the various neisserial strains was determined by search the genomes with the gonococcal Lst protein sequence (Accession number AAC44539). In strains that did not contain the Lst protein, the genomes were search with the flanking Orfs (cytochorme c or isocitrate dehygrogenase). These genes were identified in the genomes, and the flanking gene to these identified by performing a Blast search of the adjacent ORF.

Sialylation of neisserial LOS leads to increased virulence of the organism [52]. In order for an organism to sialylate its LOS, it needs to possess a sialyltransferase (Lst), and an appropriate acceptor sugar. We used the amino acid sequence for FA1090 Lst to search the various sequence databases for the presence of Lst proteins. All gonococcal and meningococcal strains possessed predicted proteins with greater than 97% identity with FA1090 Lst. Surprisingly, *N. polysaccharea* and *N. lactamica* possessed sialyltransferase proteins, while *N. cinerea* and all of the other commensal organisms lacked the gene. The data in Figure 6 indicate the genomic organization of the various strains. This data demonstrate that *N. cinerea* had deleted the coding sequence for *lst*, as it possesses flanking DNA sequences which are virtually identical to those seen in the pathogens. It is possible that the inability of *N. cinerea* to cause disease may be explained in part by the fact that it lacks Lst, as it possesses all of the genes needed to synthesize the LOS structures that have been shown to be important for virulence.

### Synthesis of the alpha chain variants

**Figure 7:**
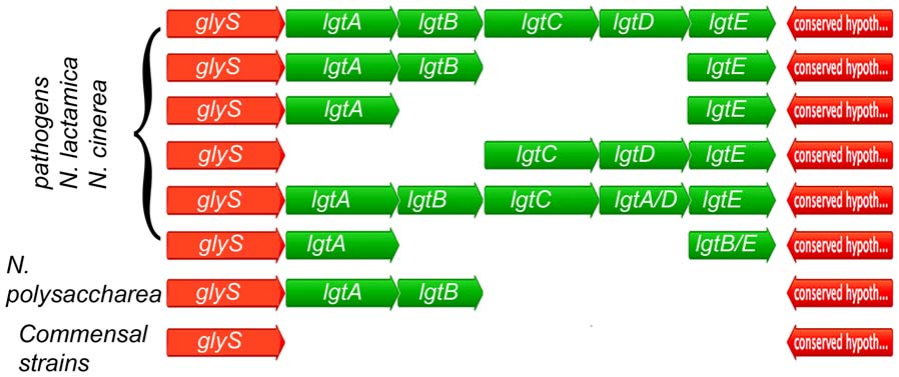
Genomic organization of the *lgt* gene cluster. The *lgt* gene cluster in *N. gonorrhoeae* strain F62 is flanked by two ORFs, glycyl tRNA synthase and a conserved hypothetical protein. Various organizational structures of the *lgt* genes were found in the pathogens, *N. lactamica*, *N. cinerea*, and *N. polysaccharea*, as indicated. All other commensal organisms tested had the glycyl tRNA synthase and a conserved hypothetical protein adjacent to each other.

In the pathogenic strains, the alpha chain has some significant genomic diversity, with deletions and/or recombination of some of the genes needed for its synthesis commonly being seen [53]. The data in Figure 7 indicate that with the exception of *N. lactamica*, *N. cinerea* and *N. polysaccharea*, none of the commensals possessed any genes found in this cluster. However, they did contain the flaking genes as linked genes. This demonstrates that the alpha chain in commensal organisms is encoded by unique genes, and should have structures different from the pathogens. This is consistent with published compositional data [38].

### Identification of regions unique to *N. sicca* 4320

**Figure 8:**
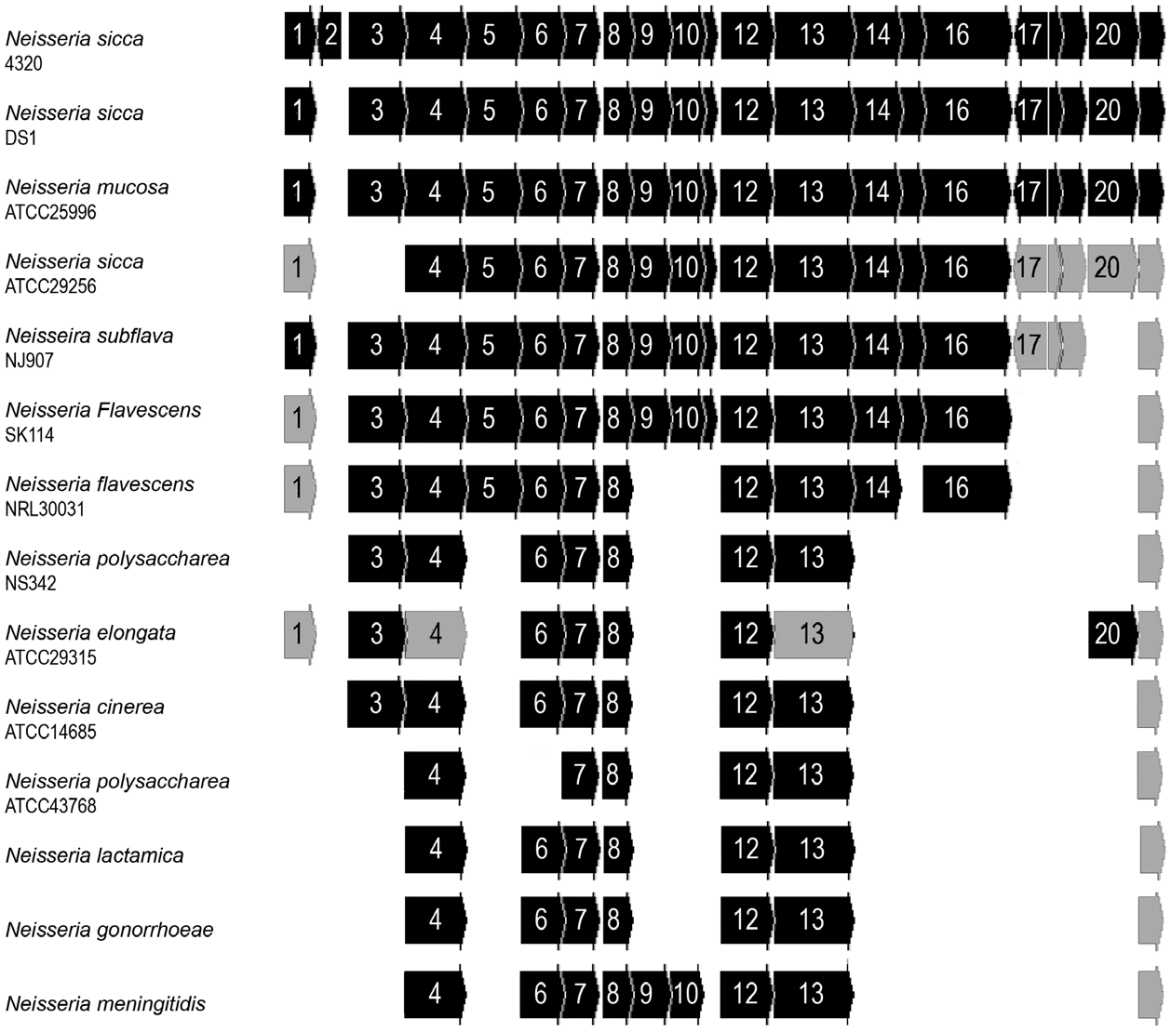
Genomic organization of the putative LPS biosynthetic gene cluster. The numbers contained within the arrows refer to open reading frames found on Contig 56 that contained genes with homology to LPS biosynthetic genes. There were 21 ORFs. ORFs 11, 15, 18, 19 and 21 are not numbered as they were too small to fit in the figure. All ORFs were compared to the organization as seen in strain *N. sicca* 4320. A solid black ORF indicates that the gene was found in the designated location. A stippled ORF indicates that the gene is present in a location elsewhere on the chromosome. Missing ORFs were not found in the test organism. All strains are as designated. If no species identification is present, it indicates that all strains from that species had the same genomic organization. The numbers located in the ORFs refer to the following proteins, which had the highest degree of similarity to proteins found in the NCBI database: 1) Rrf2-linked NADH-flavin reductase; 2) Unknown protein; 3) UDP-glucose dehydrogenase; 4) Lipopolysaccharide biosynthesis translocase; 5) Oligosaccharide repeat unit polymerase Wzy; 6) Capsular polysaccharide biosynthesis glycosyl transferase capM; 7) Glycosyl transferase); 8) UDP N-acetyl galactosaminyl transferase; 9) Putative carbamoyl phosphate synthase large subunit, short form; 10) 2-Haloalkanoic acid dehalogenase; 11) Methionyl tRNA formyl transferase; 12) Pleiotropic regulatory protein; 13) Nucleoside-diphosphate sugar epimerase; 14) Polysaccharide export protein (Wza); 15) Low-molecular weight protein-tyrosine phosphatase (Wzb); 16) Tyrosine-protein kinase (Wzc); 17) CidA-associated membrane protein CidB; 18) Holin-like protein CidA; 19) LysR family regulatory protein CidR); 20) Oxidoreductase; 21) Oxygen-insensitive NAD(P)H nitroreductase/Dihydropteridine reductase.

*N. sicca* 4320 expresses an LPS. Because it was possible that the O-repeat biosynthetic genes are only found in *N. sicca* 4320, ORFs unique to *N. sicca* 4320 were identified by comparing *N. sicca* 4320 genomic DNA with *N. meningitidis* MC58 and or the gonococcal genomes (see Table 2). One region was identified that possessed gene homologs to two glycosyl transferases, an O-antigen ligase, a chain length determinant protein, and LPS transport proteins, genes that appeared to be homologs of genes involved in LPS biosynthesis (see Figure 8). The G+C content and the codon usage seen in this region were consistent with other genomic regions found in these organisms. Further analysis of these regions indicated that the region in 4320 was largely intact in most commensal strains, but missing in the pathogens. Another region identified contained two glycosyl transferases that were unique to *N. sicca* 4320. Since *N. sicca* 4320 produces an LPS with a disaccharide O-repeat, this region would appear likely to encode for LPS biosynthesis. It is interesting to note that all strains retained the genes that appeared to be involved in LOS transport, while losing the genes needed by LPS biosynthesis. This suggests that pathogen evolution has been driven by the loss of the ability to produce LPS.

**Table 2:** Genomic similarity of putative LPS biosynthetic genes.

Organism	O-antigen polymerase/ligase	Chain length determinant protein	Putative Wza(Complex polysaccharide trasnport)	LPS biosynthesis translocase	LPS biosynthesis oxidoreductase	4-a-glucano transferase	1,4-a-glucan-branching enzyme
*N. sicca* DS1	96.3%^a^	97.6%	93.8%	97.4%	94.4%	93.9%	95.6%
*N. sicca* ATCC 29256	76.9%	85.0%	96.6%	70.8%	94.4%	94.0%	99.0%
*N. mucosa* ATCC 25996	96.6%	97.4%	93.8%	96.9%	94.1%	93.8%	96.2%
*N. subflava* NJ9703	70.1%	76.4%	78.9%	72.0%	NP^b^	76.0%	86.0%
*N. flavescens* NRL30031/H210	71.6%	76.8%	47.0%	76.8%	NP	85.6%	85.6%
*N. polysaccharea* NS342	NP	NP	NP	NP	NP	81.7%	88.3%
*N. lactamica* NS19	NP	NP	NP	NP	NP	NP	NP
*N. cinerea* ATCC 14685	NP	NP	NP	NP	NP	NP	NP
*N. meningitidis*	NP	NP	NP	NP	NP	NP	NP
*N. gonorrhoeae*	NP	NP	NP	NP	NP	NP	NP

a Percentage listed is the degree of similarity between the tested gene and the homolog found in *N. sicca* 4320.

b NP indicates that no homolog was present.

## Discussion

This study was undertaken to determine the genetic potential of the *Neisseriaceae* with respect to LPS/LOS expression. The data indicate that neisserial LOS possesses two basic core structures; one that possesses a gamma chain N-acetylglucosamine and one that contains a third heptose. The presence of the third heptose was found in strains that rarely caused disease, while the presence of the gamma chain N-acetylglucosamine was associated with pathogens. However, two commensal strains expressed a gamma chain N-acetylglucosamine, *N. lactamica* and *N. cinerea*. The inability of *N. lactamica* (most closely related to *N. meningitidis*) to cause disease relates to its inability to express a capsule. We would suggest that the inability of *N. cinerea* to cause disease is related to its inability to sialylate its LOS, however other factors may also be involved and or responsible for the avirulence of this strain.

The importance of LOS in neisserial pathogenesis is illustrated by the high degree of sequence conservation in the genes needed for LOS biosynthesis in the pathogens. It is further substantiated by the presence of homopolymeric runs of guanines in key genes involved in LOS biosynthesis, which leads to phase variable expression of LOS. We were unable to identify any homopolymeric runs, or other repeated sequences in commensal LOS biosynthetic genes, suggesting that the expression of LOS in commensal strains is invariant. The alteration in LOS structure seen in the commensal organisms suggests that the third heptose modification may play an important role in modulation LOS/LPS mediated toxicity. We are currently exploring the role of this modification on LOS and LPS, to determine its importance in immunological signaling.

## Methods

### Commensal *Neisseria* sequencing

Chromosomal DNA for *N. sicca* 4320, *N. sicca* DS1, *N. polysaccharea* 342, *N. sicca* NS19, and *N. meningitidis* 44 was isolated using the method described in Maniatis et al. [54]. The DNA sequence of each of these samples was determined by the Genomics Resource Center at the University of Maryland School of Medicine, Baltimore, MD. The sequence reads were assembled using Newbler [55] to generate a set of contigs for each commensal. The genomic sequences were submitted to NCBI: *N. sicca* 4320, GenomeProject ID #60861; *N. sicca* DS1, GenomeProject ID #60863; *N. polysacchareae* 342, GenomeProject ID #60865; *N. lactamica* NS19, GenomeProject ID #60867; and *N. meningitidis* 44, GenomeProject ID #60869.

### Mummer alignments

Unique regions of the contig sequences of *N. sicca* 4320 were determined by alignment with the contig sequences of the other identified commensals using Nucmer, a component of the Mummer software package [56]. The alignments were filtered to remove repeat alignments leaving a 1-to-1 alignment between *N. sicca* 4320 and the other sequenced commensal. Regions not aligning were identified as unique to *N. sicca* 4320.

### Geneious

The contig sequences of the commensals were imported into the program Geneious. Custom BLAST databases of each sequenced commensal were compiled to use for further analysis. Geneious was used to manage all sequences and to construct the sequence figures found in this manuscript [57].

### ORF annotation

The contig sequences were analyzed by the program Glimmer3 and putative ORFs predicted [58]. The amino acid sequences of the predicted ORFs were extracted and used as queries in BLAST searches of the various NCBI and custom databases.

### Bioinformatic BLAST screen of *Neisserial sequence*

The custom commensal BLAST databases were searched to identify sequences with similarity to glycosyltransferases. ORFs showing similarity to the sequences were used as a query in a BLAST search of the nr database to confirm similarity to putative O-repeat biosynthesis genes.
